# Valvular Insufficiency and Anasarca

**Published:** 1893-07

**Authors:** O. B. Bush

**Affiliations:** Arlington, Ga.


### VALVULAR INSUFFICIENCY AND ANASARCA.

---

Arlington, Ga., May 26, 1893.

*Editor Atlanta Medical and Surgical Journal:*

Dear Sir—Called to see Willie G., age seventeen years. Found him in a sitting posture, where he had been for some time. Pulse fast and intermittent. He has had three attacks of la grippe within eighteen months, which of course aggravated the heart disease to some extent. Found the pulse 130 to the minute; temperature normal. His weight was one hundred and ninety-three pounds. Placed him on tincture aconite, and restricted his diet to milk and eggs and oysters and crackers almost exclusively. His breathing was from thirty to forty-five to the minute. The aconite seemed to relieve this hurried breathing to some extent. Kept him on this two days. Changed treatment to bromide potash and fluid extract henbane, tincture scilla and tincture digitalis every four hours, and tincture mur. iron three times per day before meals, and bitartrate potash and sulphate magnesia three times per day after each meal; the iron and the bitartrate potash and sulphate magnesia to be taken in elder root tea. Kept patient on this for ten days, and no good derived from it. Changed treatment once more to the bromide potash and fluid extract henbane and tincture scilla and papine. Kept him on the tincture mur. iron and bitartrate potash and sulphate magnesia, and kept him on this for ten days, after which time he weighed only one hundred and forty-five pounds, and his breathing was more regular and his pulse was 100 to the minute. He was now able to come to my office, a distance of four and one-half miles. Since that time he has continued to convalesce ; but those intermissions of his pulse seem to be aggravated upon either mental or physical exercise. I have him on the bromide potash, fluid extract henbane and tincture scilla and papine, and tincture mur. iron yet, but have taken him off the bitartrate potash and sulphate magnesia, which I did not think it necessary to continue.

I think that in the course of two weeks longer it would be well for me to make an addition of tincture nox vomica, which I intend doing. I heartily recommend papine to all sufferers of pain of any source, as it has none of the objections that the other preparations of opium have.

If any one can suggest a remedy which will assist me in this case I will be much obliged. O. B. BUSH, M. D.

